# Mango (*Mangifera indica* L.) cv. Kent fruit mesocarp *de novo* transcriptome assembly identifies gene families important for ripening

**DOI:** 10.3389/fpls.2015.00062

**Published:** 2015-02-18

**Authors:** Mitzuko Dautt-Castro, Adrian Ochoa-Leyva, Carmen A. Contreras-Vergara, Magda A. Pacheco-Sanchez, Sergio Casas-Flores, Alejandro Sanchez-Flores, David N. Kuhn, Maria A. Islas-Osuna

**Affiliations:** ^1^Laboratorio de Genética y Biología Molecular de Plantas, Centro de Investigación en Alimentación y DesarrolloHermosillo, Sonora, Mexico; ^2^Instituto Nacional de Medicina Genómica, Unidad de Genómica de Poblaciones, Aplicada a la Salud, Facultad de Qumica UNAM, DelegaciónTlalpan, Mexico DF; ^3^Laboratorio de Genómica Funcional y Comparativa, División de Biología Molecular, Instituto Potosino de Investigación Científica y TecnológicaSan Luis Potosí (SLP), Mexico; ^4^Unidad Universitaria de Secuenciación Masiva de DNA, Instituto de Biotecnología/Universidad Nacional Autónoma de MéxicoCuernavaca, Morelos, Mexico; ^5^United States Department of Agriculture – Agricultural Research Service, Subtropical Horticulture Research StationMiami, FL, USA

**Keywords:** *Mangifera indica* L., mesocarp, fruit ripening, transcriptome, cell wall hydrolytic enzymes, ethylene, fruit quality

## Abstract

Fruit ripening is a physiological and biochemical process genetically programmed to regulate fruit quality parameters like firmness, flavor, odor and color, as well as production of ethylene in climacteric fruit. In this study, a transcriptomic analysis of mango (*Mangifera indica* L.) mesocarp cv. “Kent” was done to identify key genes associated with fruit ripening. Using the Illumina sequencing platform, 67,682,269 clean reads were obtained and a transcriptome of 4.8 Gb. A total of 33,142 coding sequences were predicted and after functional annotation, 25,154 protein sequences were assigned with a product according to Swiss-Prot database and 32,560 according to non-redundant database. Differential expression analysis identified 2,306 genes with significant differences in expression between mature-green and ripe mango [1,178 up-regulated and 1,128 down-regulated (*FDR* ≤ 0.05)]. The expression of 10 genes evaluated by both qRT-PCR and RNA-seq data was highly correlated (*R* = 0.97), validating the differential expression data from RNA-seq alone. Gene Ontology enrichment analysis, showed significantly represented terms associated to fruit ripening like “cell wall,” “carbohydrate catabolic process” and “starch and sucrose metabolic process” among others. Mango genes were assigned to 327 metabolic pathways according to Kyoto Encyclopedia of Genes and Genomes database, among them those involved in fruit ripening such as plant hormone signal transduction, starch and sucrose metabolism, galactose metabolism, terpenoid backbone, and carotenoid biosynthesis. This study provides a mango transcriptome that will be very helpful to identify genes for expression studies in early and late flowering mangos during fruit ripening.

## INTRODUCTION

Fruit ripening is a complex biochemical and physiological process where modifications in cell wall and secondary metabolism lead to changes in macroscopic parameters such as appearance, texture, flavor, and aroma. The molecular basis of fruit ripening has been widely studied by traditional biochemistry, genetics and molecular biology in fruits like tomato, papaya, strawberry, apple, peach, among others (Gapper et al., 2013). Mango (*Mangifera indica* L.), a member of the Anacardiaceae family, is the second most important tropical fruit crop in the horticulture industry worldwide (Singh et al., 2013). So far, gene expression changes in mango mesocarp have been studied only for specific genes to provide hints into the ripening process. RNA sequencing (RNA-seq) is a useful technology to measure global changes in transcription (Marguerat and Bähler, 2010). RNA-seq has been used to understand the ripening process in fruits like Chinese bayberry, watermelon and orange (Guo et al., 2011; Feng et al., 2012; Yu et al., 2012). Therefore, the RNA-seq of mango mesocarp can provide insights about specific gene expression patterns for mature-green and ripe mango.

Mango is a climacteric fruit, and the expression of ethylene biosynthesis genes like ACC synthase (*ACS*) and ACC oxidase (ACO) are predicted to change during ripening, as previously shown in tomato. Studies in tomato show that *LeACS2* and *LeACO1* dominate gene expression during climacteric ethylene production. Other important ethylene-related genes are membrane receptors involved in regulation of secondary metabolites (White, 2002). The *Arabidopsis* ethylene receptor family is comprised of five members divided into two subfamilies: ETR1 and ERS1, subfamily I; ETR2, ERS2, and EIN4, subfamily II (Bleecker, 1999). However, it is necessary to identify those genes and pathways during mango mesocarp ripening.

Firmness is a key post-harvest quality attribute, and it dictates commercialization strategies, since it must reach the consumer in 2 weeks at the most. Firmness loss is due to the action of cell wall hydrolytic enzymes such as polygalacturonases (PGs), pectin methyl esterases (PMEs), pectate lyases (PLs), α-galactosidase (α-GAL), β-galactosidases (β-GAL), glucosidases (Glu), among others (Goulao and Oliveira, 2008). There is also a key physical process that is involved in fruit softening and is due to the action of expansins, which are cell wall proteins that loosen cellulose structure without any hydrolytic activity (McQueen-Mason and Cosgrove, 1995).

Color changes during fruit ripening include the conversion of chloroplasts to chromoplasts. As a result of the loss of photosynthetic capacity of the chloroplasts, thylakoid structures become sites for the accumulation of carotenoids in the fruit cells (Klee and Giovannoni, 2011). The pigment accumulation in mango fruit is cultivar-dependent, but in general, mango has a high content of carotenoids in mesocarp tissue responsible for the intense yellow color (Singh et al., 2013). The enzymes involved in carotenogenesis change during fruit ripening. For example, phytoene synthase (PSY) and carotenoid beta-hydroxylase-1 (CHYB1) accumulate at the tomato breaker stage compared to the red-ripe stage, leading to high levels of lycopene (Smita et al., 2013). The fruit flavor is not always directly related to their sugar content. Volatile compounds like monoterpenes, sesquiterpenes, terpenoids, carotenoids, and amino acids are also critical for ripe-mango flavor (El Hadi et al., 2013). The terpene hydrocarbons are important factors for mango flavor in cultivars like “Kent,” “Keitt,” and “Tommy Atkins” (Singh et al., 2013). Terpene synthases that convert prenyl diphosphates to terpenes were identified by RNA-seq in *Solanum lycopersicum* stem trichomes and were expressed in several tissues and enriched in some others (Bleeker et al., 2011).

In order to understand the mango ripening process, it is very important to know the gene families associated with the quality parameters mentioned above as well as their expression patterns. Therefore, the objective of this study was to obtain the transcriptome from mature-green and ripe mango mesocarp in order to identify differentially expressed transcripts involved in mango ripening for ethylene production, softening, sugar synthesis, volatile compounds, and color pigments to elucidate and to investigate in depth the ripening process in this fruit.

## MATERIALS AND METHODS

### PLANT MATERIAL AND RNA PREPARATION

Mango (*M. indica* L.) fruit cultivar “Kent” was harvested at the National Institute of Forestry, Agricultural and Veterinary Research (INIFAP) research station located in Navojoa, Sonora, Mexico (27°03′49.33′′ N and 109°30′11.42′′ W). This is a commercial mango variety, is not an endangered species and no permit is required for sampling. Samples were homogeneous in shape, size and physiological maturity and transported to the laboratory. Fruits were disinfected with chlorinated water and stored at 20°C up to 10 days.

Total RNA was isolated from mango mesocarp tissue pulverized with liquid-nitrogen. Sampling was done at day 1 (mature-green mango) and day 10 (ripe mango), using two individual fruits for each ripening stage. RNA was isolated as previously described Lopez-Gomez and Gómez-Lim (1992), and then it was treated with RNase-free DNase I (Roche) to eliminate contaminant genomic DNA. The RNA quantity was estimated using a Nano-Drop ND-1000 UV-Vis spectrophotometer at 260 nm (Nano Drop Technologies Inc., Wilmington, DE, USA). RNA integrity was analyzed by 1% agarose gel electrophoresis under denaturing conditions and with the 2100 Bioanalyzer system (Agilent Technologies, Santa Clara, CA, USA).

### RNA-seq LIBRARY PREPARATION, SEQUENCING, *DE NOVO* ASSEMBLY, AND DIFFERENTIAL EXPRESSION ANALYSIS

Two mature-green and two ripe mango were selected to prepare four independent cDNA libraries using the Illumina TruSeq RNA sample preparation Kit v2 following the manufacturer instructions. These four libraries were sequenced using the Genome Analyzer GAIIx II (Illumina) at the Institute of Biotechnology-UNAM (Cuernavaca, Morelos, Mexico) with a configuration for pair end reads with a 72 bp read length.

The mango mesocarp transcriptome was assembled with the Trinity software (Grabherr et al., 2011). A pooling strategy was used only for the transcriptome *de novo* assembly in order to increase the chances of reconstructing low expressed transcripts. Each set of reads from the two biological replicates of mature-green and ripe mango were mapped back to those transcripts using the Bowtie aligner with some Perl script provided with the Trinity pipeline.

To calculate the differential expressed (DE) genes between the mature-green and ripe mango the transcripts from each library were normalized using the RPKM method.

The initial reads for each replicate and each condition were mapped back to the transcriptome assembled by using the “align_and_estimate_abundance.pl” Perl script from the Trinity pipeline. Each condition with its replicates were mapped and counted separately as described previously. Using the RSEM counts for each sample, a merged table was generated using the “abundance_estimates_to_matrix.pl” Perl script that is also included in the Trinity pipeline. For the differential expression analysis we used the “run_DE_analysis.pl” script with the merged table. The “run_DE_analysis.pl” script takes a read count cut-off of 10 reads after adding the values of all replicates and conditions (min_rowSum_counts 10 [default]). The DE genes were filtered using a False Discovery Rate (FDR) cut-off line *p* ≤ 0.05.

### TRANSCRIPT ANNOTATION AND PATHWAY ANALYSIS

To identify homologous proteins, all mango “Kent” mesocarp deduced amino acid sequences were BLASTed against the non-redundant (NR) and Swiss-Prot database using the Blast2GO software (Conesa and Götz, 2008). The E-value cut-off was set at 1.0E^-3^. Mango proteins were functionally mapped to GO terms and annotated with the following parameters: E-Value-Hit-Filter: 1.0E^-3^. Annotation cut-off: 55; GO weight: 5; Hsp-Hit Coverage cut-off: 0. Statistically enriched GO terms represented in the DE genes were also identified using Blast2GO setting with the term filter value to *p* ≤ 0.05 and the term filter mode FDR. Mango mesocarp proteins were associated with protein families and domains with InterProScan. Additionally, the annotation of Clusters of Orthologous Groups of proteins (COG) was obtained (Tatusov et al., 2000).

The transcripts were analyzed with the Kyoto Encyclopedia of Genes and Genomes (KEGG) pathway database (Kanehisa and Goto, 2000). The KEGG Automatic Annotation Server (KAAS, http://www.genome.jp/kaas-bin/kaas_main?mode=partial) was employed to map KEGG pathways, using the BBH (bi-directional best hit) method to assign orthologs. The percentages of coverage were predicted by contrast to the KEGG pathways of *Citrus sinensis* database.

### VALIDATION OF DIFFERENTIAL GENE EXPRESSION BY REAL-TIME TRANSCRIPTION PCR (qRT-PCR) ANALYSIS

First-strand cDNA was synthesized from 5 μg of DNA-free RNA (from the same mature-green and ripe mango fruits that were used for RNA-seq) using Super Script III reverse transcriptase (Invitrogen) according to the manufacturer’s protocol. The cDNA was adjusted to a 100 ng/μl concentration and it was diluted in an 1:5 ratio, to evaluate the primers of the target genes and the reference gene glyceraldehyde-3-phosphate dehydrogenase (*GAPDH*). qPCR was done using iTaq Universal SYBR Green Supermix (BIO-RAD).

The expression of 10 genes that were DE during mango ripening according to the transcriptome data were PCR-amplified in triplicate in reactions that included 5 μl of cDNA template (4–20 ng), 10 μL of iTaq Universal SYBRGreen Supermix, 1 μL of 5 μM of each sense and antisense primer and RNase-free water to a 20 μL final volume. Specific primers are listed in Table S1. The PCR was done in a Step-One^TM^ Real-time PCR System (Applied Biosystems Inc, Foster City, CA, USA). Amplification conditions were 95°C for 10 min and 40 cycles of 95°C for 15 s and 60°C for 1 min. PCR specificity was confirmed by constructing a melt curve after amplification in a range from 95°C for 15 s, 60°C for 1 min and 95°C for 15 s. Non-template controls were also included. The results were normalized to the *GAPDH* expression levels and analyzed with the 2^-ΔΔCT^ method (Livak and Schmittgen, 2001).

Statistical analysis was performed using one-way ANOVA, with a significance level of *p* < 0.05, using the NCSS (2007) software. A linear regression analysis was done to obtain the correlation between transcript abundance assayed by qRT-PCR and the transcription profile revealed by RNA-seq data. The correlation coefficient (*R*) was obtained by non-parametric analysis (Spearman).

## RESULTS AND DISCUSSION

### MANGO RNA-seq AND *DE NOVO* ASSEMBLY

A total of 234,310,610 Illumina reads from both, mature-green and ripe conditions were obtained with a total of 16,870,363,920 bases (16 Gb). The FastQC program was used to evaluate the quality of the reads and low-quality or adapter sequences were removed. After trimming and discarding low-quality reads, 67,682,269 clean reads (4,873,123,368 pb) were used. The GenBank accession number for those reads is SRP045880. After Trinity assembly 80,969 transcripts were obtained with a mean length of 836 bp and N_50_ of 1,456 bp. The largest transcript was 8,713 nucleotides long. Transcripts were assembled into 52,948 putative unigenes of which 33,142 had an open reading frame (ORF). Detailed information about the assembly statistics are presented in **Table 1**. A leaf transcriptome of mango “Langra” was reported (Azim et al., 2014) where 85,651 transcripts were obtained with a mean size of 536 bp resulting in 30,509 unigenes. In this study, we obtained 22,439 more unigenes and a significantly higher mean transcript length. The pooled transcriptomes from mango “Zill” pericarp and pulp had 124,002 transcripts with a mean size of 838 bp resulting in 54,207 unigenes (Wu et al., 2014a) essentially similar to our results.

**Table 1:** Summary of mango mesocarp RNA-seq and *de novo* assembly.

Sequence	Value
Total bases	4,873,123,368
Total RNA-seq reads	67,682,269
Total transcripts	80,969
Average length of transcripts	835.90
Largest transcript	8713
N_50_ length of transcripts	1456, *n* = 14912
Putative unigenes	52,948
Transcripts with a ORF	33,142

### ANNOTATION AND FUNCTIONAL CHARACTERIZATION

**FIGURE 1:**
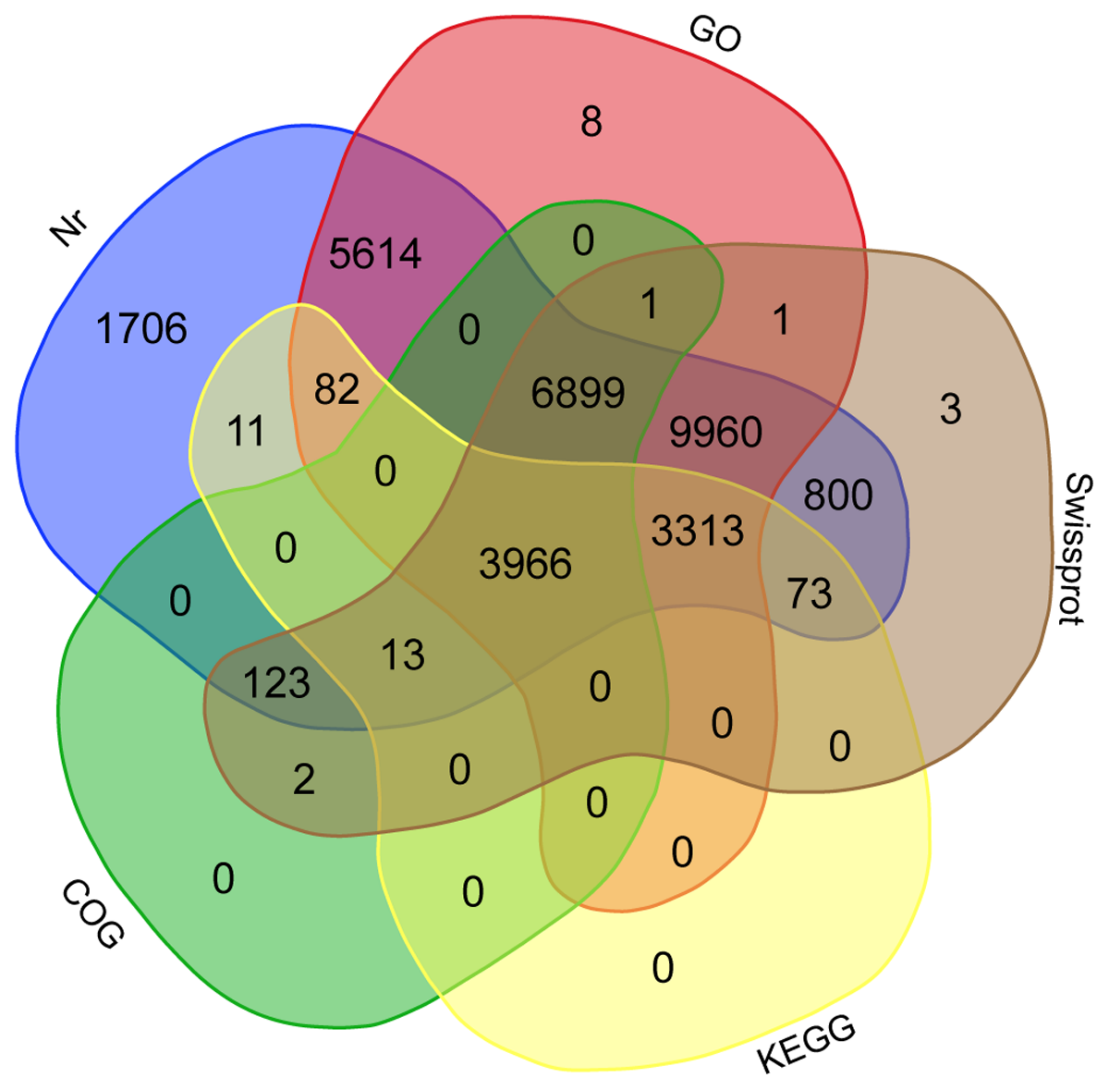
**Venn diagram of mango shared unigenes.** Annotation was done according to the NCBI non-redundant (Nr), Swiss-Prot, COG, and KEGG databases and classified into Gene Ontology (GO). Overlapped unigenes are indicated in the intersections.

**FIGURE 2:**
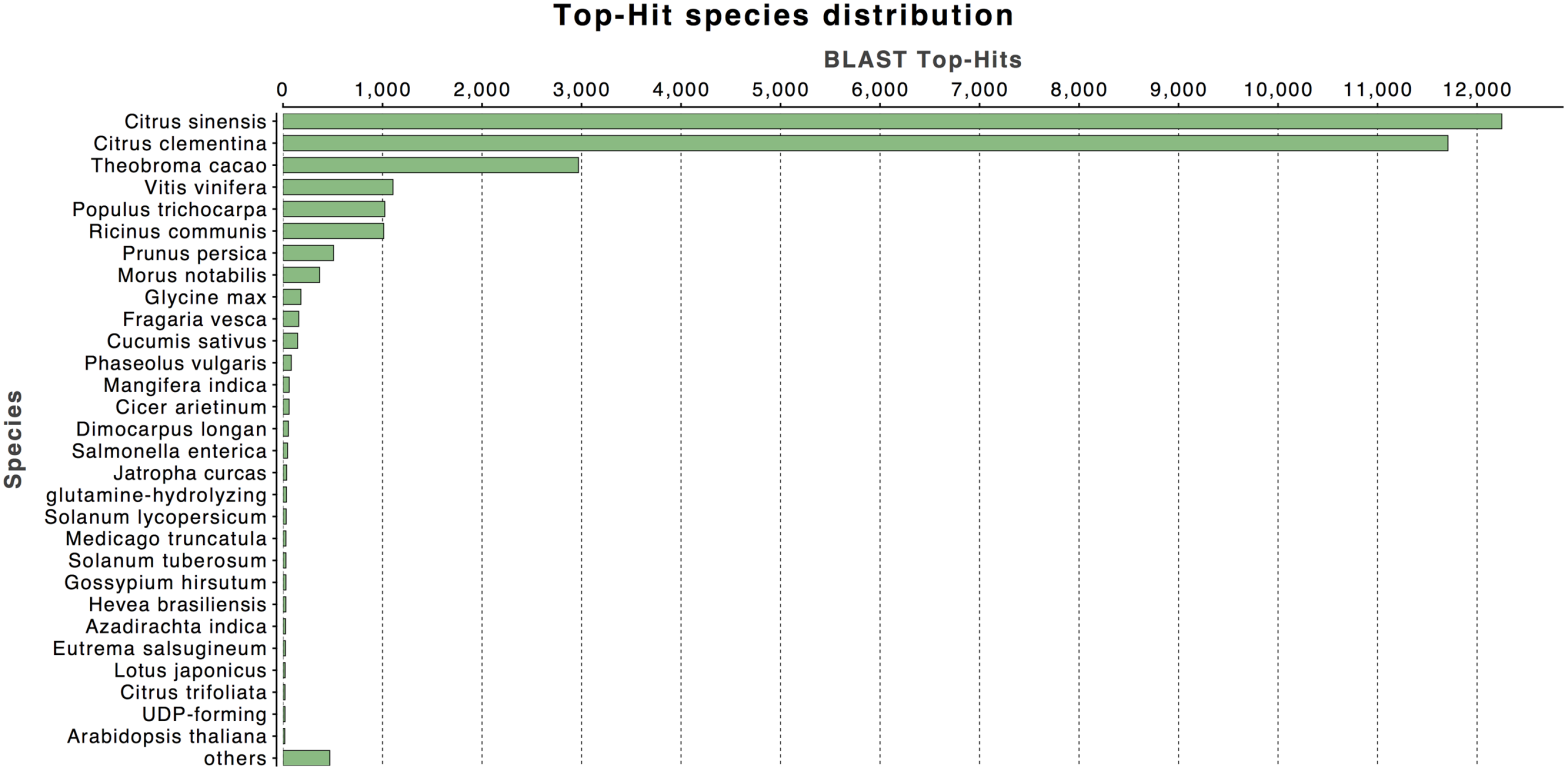
**Top-hit species taxonomic distribution.** Number of Unigenes matching the 30 top species using BLASTx in the NR database.

The annotation of assembled sequences was performed using the Swiss-Prot and NCBI non-redundant databases and the results are shown in **Figure 1**. According to the Swiss-Prot database, 25,154 genes were fully annotated, 3,905 were partially annotated (Psignal, Pfam, COG, among others) and for 5,264 there was no annotation. In contrast, the results of the annotation performed with the NR database suggest that 32,560 deduced proteins (98.2%) showed significant BLASTp matches with amino acid sequences deposited in the nr database (cut-off E-value of 10^-3^). *Citrus* protein sequences were the most commonly matched with 36.9% (12,249 sequences) of the 33,142 proteins matched with *C. sinensis* followed by 35.3% with *Citrus clementina* (11,708 amino acid sequences; **Figure 2**). A phylogenetic study of mango chloroplast DNA (cpDNA) reported that *C. sinensis* was closely related to *M. indica* (Azim et al., 2014). *M. indica* L., meanwhile only presented hits for 62 amino acid sequences, as little publicly available sequence information previously existed for this species.

The predicted mango genes were classified into the three Gene Ontology (GO) categories: Cellular Component, Biological Process, and Molecular Function. Based on sequence homology, 29,844 mango unigenes (**Figure 1**) were categorized into 81,315 functional groups. The Cellular Component category-contained 10,999 unigenes with the most highly represented terms being “cell part” (9,002), “membrane-bounded organelle” (5,332) and “protein complex” (2,448; Figure S1). The Biological Process category contained 16,055 unigenes with the more highly represented GO terms including “organic substance metabolic process” (10,261), “primary metabolic process” (9,805) and “cellular metabolic process” (9,557; Figure S2). The Molecular Function category contained 21,331 unigenes with the most frequent GO terms being “organic cyclic compound binding” (7,686), “heterocyclic compound binding” (7,677), and “ion binding” (6,890; Figure S3). In the mango leaf transcriptome (Azim et al., 2014), similar GO terms at similar frequencies to the results of this work were observed, where the terms “cell part” for cellular component, “cellular process” for biological process and “binding” for molecular function, were their most representative GO terms.

According to the COG database 11,004 proteins showed a functional annotation. The most representatives COG annotations were Serine/threonine protein kinase (COG0515) with 1,318 proteins. These kinases catalyze the phosphorylation of serine or threonine residues on target proteins by using ATP as phosphate donor, which is a way to regulate the function of the target protein (UniProt Consortium, 2014). Followed by FOG: WD40 repeat (COG2319) with 301 proteins, which are implicated in a variety of functions that include signal transduction, regulation of transcription and cell cycle control, among others (Stirnimann et al., 2010). And by leucine-rich repeat (LRR) protein (COG4886) with 190 proteins, which are proteins whose main function appears to be to provide a versatile structural framework for the formation of protein–protein interactions (Kobe and Kajava, 2001).

Transcripts were also analyzed in the KEGG database and a total of 7,458 unigenes (**Figure 1**) were assigned to 327 KEGG pathways. Tables S2 and S3 show 30 metabolic pathways of genes for both the total mango transcriptome and the differentially expressed subset. The most significant pathways with regard to the number of hits from the whole transcriptome data were “biosynthesis of amino acids,” “ribosome” and “RNA transport,” different to the results of the mango “Zill” transcriptome, who reported that “metabolic pathways,” “biosynthesis of secondary metabolites” and “plant-pathogen interactions” were the maps with the highest transcripts representation (Wu et al., 2014a). In the other hand, 3,966 unigenes were annotated in all the databases, including NR, Swiss-Prot, GO, COG and KEGG (**Figure 1**).

Fruit ripening is a complex process that involves physiological and biochemical modifications associated with metabolic pathways, like the conversion of starch to sugars, changes in the biosynthesis and accumulation of pigments, biosynthesis of flavor and aromatic volatiles and remodeling of the structure of cell wall (Goulao and Oliveira, 2008). **Table 2** shows five major pathways associated with fruit ripening, the number of gene products involved in each and the percentage of gene products of these pathways identified from the transcriptome or the differentially expressed subset with respect to the *C. sinensis* database. The metabolic pathways are: plant hormone signal transduction (Figure S4), starch and sucrose metabolism (Figure S5), galactose metabolism (Figure S6), terpenoid backbone biosynthesis (Figure S7) and carotenoid biosynthesis (Figure S8).

**Table 2:** Some important metabolic pathways involved in mango fruit ripening according to the Kyoto Encyclopedia of Genes and Genomes (KEGG) *Citrus sinensis* database.

KEGG pathway	Number of genes in total transcriptome/percentage of coverage (%)	Number of DE genes/percentage of coverage (%)
Plant hormone signal transduction	36 (85.70)	20 (47.61)
Starch and sucrose metabolism	37 (100)	14 (49.99)
Galactose metabolism	16 (97.77)	6 (55.99)
Terpenoid backbone biosynthesis	30 (100)	7 (22.58)
Carotenoid biosynthesis	15 (91.11)	6 (51.11)

### DIFFERENTIAL GENE EXPRESSION DURING MANGO RIPENING

Using the edgeR pipeline included with the Trinity software, a total of 2,306 genes were identified as differentially expressed between the mature-green and ripe mango stages, representing 6.9% of total unigenes with a predicted ORF (*FDR* < 0.05). From these, 1,178 were up-regulated and 1,128 were down-regulated (Figure S9).

As was expected in a climacteric fruit, there was a wide range of genes up-regulated during mango ripening, such as carbohydrate catabolism, sucrose and ethylene biosynthesis among others. Genes encoding 1-aminocyclopropane-1-carboxylate (ACC) oxidase, PME, PG, PL, endoglucanase, expansin, β -galactosidase, α -galactosidase, rhamnogalacturonate lyase (RGL), and β -amylase among others were expressed at higher levels at ripe mango and are listed in **Table 3**. These genes are generally encoded in multiple copies in plants. Family members of PGs, PLs, β -galactosidases and other unigenes identified in this transcriptome will be very helpful in future studies where their expression profiles at different developmental stages will be assayed at the transcriptional and translational level.

**Table 3:** List of some up-regulated genes involved in major processes associated with fruit ripening according to RNA-seq data (*FDR* ≤ 0.05).

Gene identifier	Function	Gene description	Fold-change (Log_2_)	FDR
comp59876	Ethylene biosynthesis and signaling	1-aminocyclopropane-1-carboxylate oxidase (ACO)	7	6.72E-09
comp35681	Ethylene biosynthesis and signaling	1-aminocyclopropane-1-carboxylate oxidase (ACO)	2	0.0032
comp36021	Ethylene biosynthesis and signaling	1-aminocyclopropane-1-carboxylate oxidase (ACO)	4	1.30E-11
comp27110	Ethylene biosynthesis and signaling	Ethylene-responsive transcription factor (ERF)	7	1.45E-11
comp45079	Cell wall metabolism	Pectin methyl esterase (PME)	8	1.75E-12
comp28658	Cell wall metabolism	Polygalacturonase (PG)	12	3.00E-06
comp39571	Cell wall metabolism	Polygalacturonase (PG)	9	1.02E-06
comp35446	Cell wall metabolism	Polygalacturonase (PG)	10	5.09E-08
comp34301	Cell wall metabolism	Polygalacturonase (PG)	10	1.93E-06
comp28658	Cell wall metabolism	Polygalacturonase (PG)	12	3.00E-06
comp63384	Cell wall metabolism	Pectate lyase (PL)	11	4.42E-13
comp28245	Cell wall metabolism	Pectate lyase (PL)	6	8.37E-07
comp49359	Cell wall metabolism	Pectate lyase (PL)	5	7.62E-12
comp21883	Cell wall metabolism	Pectate lyase (PL)	2	4.42E-13
comp41701	Cell wall metabolism	Beta-galactosidase (β-GAL)	12	0.0002
comp41168	Cell wall metabolism	Beta-galactosidase (β-GAL)	10	0.0001
comp35981	Cell wall metabolism	Beta-galactosidase (β-GAL)	9	0.0001
comp33387	Cell wall metabolism	Endoglucanase (GUN)	9	0.0001
comp17443	Cell wall metabolism	Endoglucanase (GUN)	10	0.0002
comp46653	Cell wall metabolism	Alpha-galactosidase (α -GAL)	2	0.0165
comp51667	Cell wall metabolism	Expansin (EXP)	5	9.90E-09
comp32472	Cell wall metabolism	Expansin (EXP)	17	8.22E-20
comp35294	Cell wall metabolism	Expansin (EXP)	6	5.08E-08
comp25112	Cell wall metabolism	Rhamnogalacturonate lyase (RGL)	5	2.82E-13
comp44364	Isoprenoid biosynthetic process	Isopentenyl-diphosphate delta-isomerase (IDI)	2	0.0084
comp48152	Isoprenoid biosynthetic process	Farnesyl pyrophosphate synthase (FPPS)	2	0.0001
comp48107	Isoprenoid biosynthetic process	Geranylgeranyl pyrophosphate synthase (GGPS)	8	2.59E-16
comp45134	Sucrose metabolic process	Sucrose synthase 7 (SuSy)	7	1.72E-16
comp42184	Carotenoid metabolic process	Lycopene isomerase (CRTISO)	4	0.0014
comp45152	Carotenoid metabolic process	Beta-carotene 3-hydroxylase (CHYB)	4	0.0061
comp44532	Polysaccharide catabolic process	Beta-amylase (BAM)	3	1.02E-06
comp39992	Polysaccharide catabolic process	Beta-amylase (BAM)	2	0.0001

**FIGURE 3:**
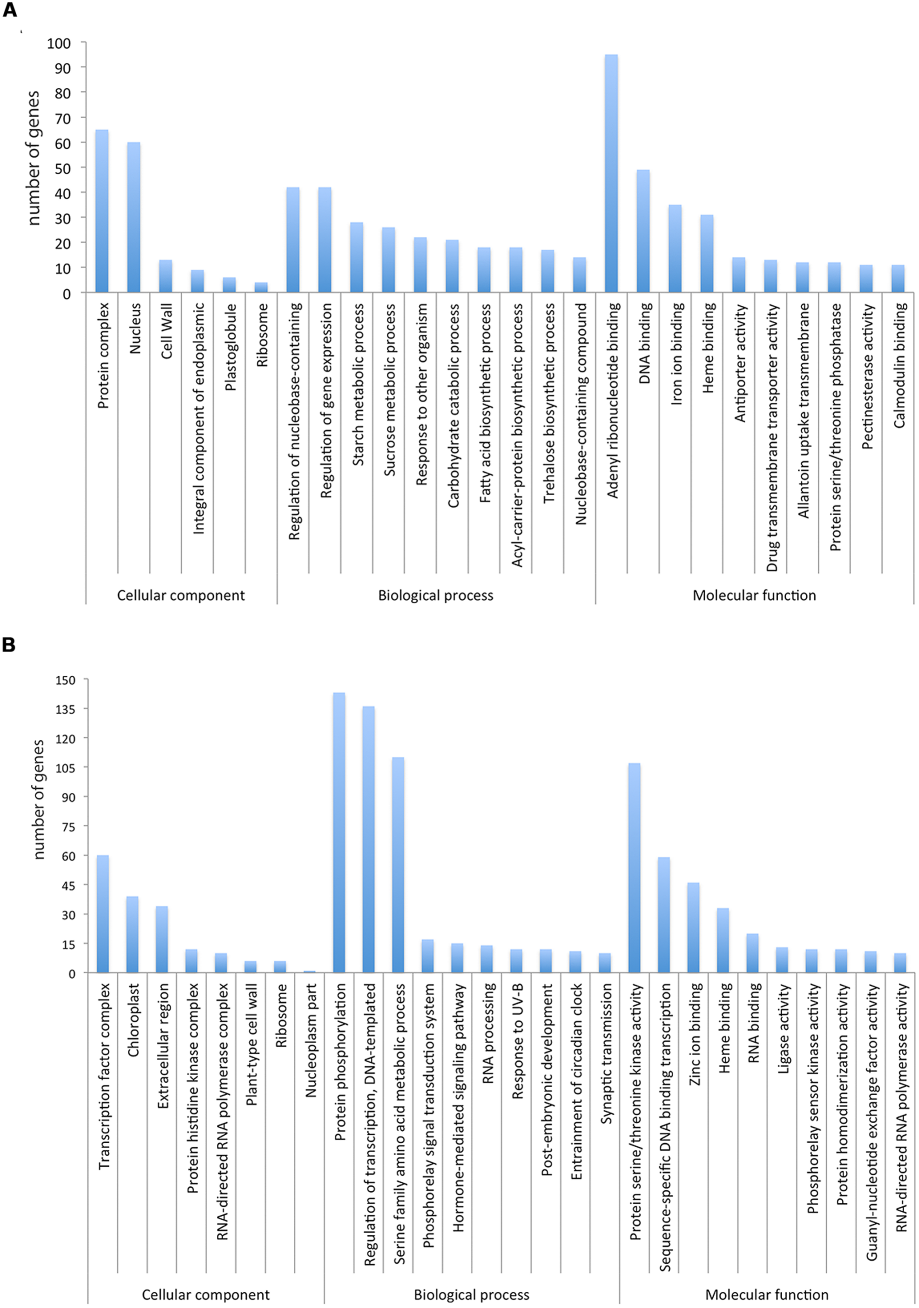
**(A)** Significantly enriched GO terms in the up-regulated transcripts ranked according to the *p*-value and number of genes. **(B)** Significantly enriched GO terms in the down-regulated transcripts ranked according to the *p*-value and number of genes.

We determined if particular GO terms were enriched in the DE genes as compared to the complete transcriptome. **Figure 3** shows the significantly enriched terms ranked according to the *p*-value and number of genes. Some enrichment of GO terms of up-regulated genes (**Figure 3A**) are associated to fruit ripening like “cell wall” and “integral component of endoplasmic reticulum membrane,” “starch metabolic process” and “sucrose metabolism.” Similar up-regulation of genes associated with “cell wall” and the “starch metabolic process” during fruit ripening has been previously observed (Feng et al., 2012; Wu et al., 2014b). In addition, genes involved in other important biological processes such as “regulation of gene expression,” “carbohydrate catabolic process” and “DNA binding” were also identified. Some enrichment of GO terms for down-regulated genes (**Figure 3B**) was associated to, “protein phosphorylation,” “regulation of transcription” and “protein serine/threonine kinase activity.”

#### Ethylene biosynthesis and signaling pathway genes

Four genes encoding the ethylene biosynthesis enzyme (ACC) oxidase and two encoding ACC synthase were obtained in this study. Three ACC oxidase genes were up-regulated in ripe mango (comp35681, comp36021, comp59876) with 2, 4, and 7-fold changes, respectively. The expression of the two ACC synthase genes did not change. Both ACC oxidase and ACC synthase are encoded by a multigene family of five and nine members, respectively, in tomato (Nath et al., 2006).

Ethylene signaling is a plant hormone signal transduction pathway. Several ethylene response genes have been identified in *Arabidopsis* (Bleecker, 1998; Schaller and Kieber, 2002) and tomato (Lashbrook et al., 1998; Tieman and Klee, 1999). In this work we have identified four ethylene receptor transcripts: *ETR1* (comp46286) and *ETR2* (comp47044), that remain constant during fruit ripening, and *ERS1* (comp32379) and *EIN4* (comp34039) that showed a decrease in their expression levels.

Other components of the ethylene signaling pathway, such as the constitutive triple response CTR1 (comp43157), which was down-regulated (-2-fold) and eleven ethylene-responsive transcription factor (ERF) were found in this transcriptome, where one (comp27110) was up-regulated (7-fold), seven were down-regulated (comp18706, comp26333, comp34532, comp39082, comp40325, comp42703, and comp45406) in mango ripe and three did not changed (comp24020, comp41521, comp45323). In a previous report (Wu et al., 2014b), one *ERF* showed a decrease in its expression levels during orange ripening.

Regulator proteins EIN2 (comp43660), EIN3 (comp47416), and EIL3 (comp41806) were also identified and their levels remained constant. EIN3 proteins are controlled by two F-box proteins (EBF1/2), which are SKP–Cullin–F-box (SCF) E3 ligases targeting EIN3 and EIL for 26S proteosome degradation (Guo and Ecker, 2003). Two EBF (ethylene binding F-Box proteins) sequences were found in this study, EBF1 (comp45329) was up-regulated (2-fold) and EBF2 (comp44836) was down-regulated (-2-fold) in ripe mango.

#### Genes associated with firmness

Mango fruit ripening involves changes in cell wall structure and degradation of lamella media, which are mainly composed by pectin, as well as loss of starch, which result in loss of firmness (Yashoda et al., 2006; Cárdenas-Coronel et al., 2012). Gene families encoding for PGs, PMEs, PLs, RGL, α-GAL, β-GAL and β-glucosidases (β-Glu) that degrade cell wall components, as well as α and β-amylases that degrade starch were DE in this work (*FDR* < 0.05).

In this study, nine *PG* unigenes were obtained and six of them (comp28658, comp34301, comp35446, comp39571, comp39826, comp39773) were up-regulated in ripe mango, with 12, 10, 10, 9, 2, and 2-fold changes, respectively. One *PG* was down-regulated (comp26280) with a -4 fold. These results suggest that PG expression is developmentally regulated and that most of the PGs act in late phase of ripening but they could act also at the beginning. In papaya (*Carica papaya*), Fabi et al. (2014), reported that *cpPG1* plays a central role during pulp softening. This *cpPG1* presented higher expression levels compared with other three papaya *PG*. Comparing the amino acid sequences of cpPG1 against mango PG, those encoded in comp35446 and comp28658, were 68 and 67% identical, respectively. The up-regulation of these two PG (comp35446 and comp28658) in ripe mango could imply their role in pulp softening. In banana (*Musa acuminata*), four genes encoding PG associated with fruit ripening were also reported (Mehar and Pravendra, 2005). Comparison of amino acid sequences of these PGs and mango PGs showed a high percentage of identity (up to 62%) and their expression patterns were also similar. In mango cultivars “Alphonso” and “Dashehari,” three PG isoforms that showed an increase in their activity during fruit ripening were reported (Prasanna et al., 2006; Singh and Dwivedi, 2008).

Pectin, formed by galacturonic acid with α-1,4 linkages, generally is highly methyl-esterified and must be de-esterified by PME to be later processed by PG (Brummell and Harpster, 2001). Seven *PME* cDNAs were found in this transcriptome, six of them (comp45079, comp198908, comp27472, comp173846, comp37143, comp37279) were up-regulated with 8, 7, 6, 5, 3, and 2-fold changes, respectively and one (comp43676) was down-regulated (-2-fold) in ripe mango. These results suggest that PME expression is differentially regulated during mango ripening.

The abundance of four *PL* (comp63384, comp28245, comp49359, comp21883) transcripts increased during mango ripening with 11, 6, 5, and 2-fold changes, respectively. In a previous study with mango “Dashehari,” a PL gene associated to fruit ripening was reported and its expression levels increased in ripe mango (Chourasia et al., 2006). This PL gene is similar to the PL gene (comp21883) reported in this transcriptome (both are 99% identical in their amino acid sequences). In banana, two different *PL (Pel I and Pel II)* cDNAs associated with fruit ripening have been reported. Both *PL* presented higher levels of expression in ripe bananas (Marin-Rodriguez et al., 2003).

In this study, only one *RGL* (comp25112) was obtained and it was DE in ripe mango (5-fold). It is known that RGL degrades pectin type rhamnogalacturonan I, but little is known about how it acts in fruit cell walls. In potato (*Solanum tuberosum* L.) plants transformed with fungal *RGL*, the resulting tubers had cells with an altered morphology and their cell wall contents were easier to extract than those from wild-type plants, suggesting the importance of this enzyme (Oomen et al., 2002). Recently, in strawberry (*Fragaria x ananassa*) an RGL gene was reported to be up-regulated in receptacle in ripe fruit and it was related to the degree of firmness of fruit according to genetic linkage association analysis (Molina-Hidalgo et al., 2013). This strawberry RGL enzyme is 57% identical in amino acid sequence to the mango RGL (comp25112) from this transcriptome. This enzyme could be important in softening and it will be interesting to uncover its function.

Two of the main pectin deglycosylating enzymes that participate in this process are α-GAL and β-GAL (Goulao and Oliveira, 2008). In this study, four α-*GAL* transcripts were identified and three of them (comp38455, comp42509, comp45738) did not changed expression levels during ripening; while one of them (comp46653) was up-regulated (2-fold change). Nine β-GAL genes were identified and three (comp41701, comp41168, comp46653) were up-regulated with fold-changes of 12, 10, and 2, respectively, in ripe mango; while five β*-GAL* transcripts (comp42509, comp250695, comp44177, comp45738, comp44177) expression levels did not changed in ripe mango. Among the glycosidases from mango, β-GAL appeared to be the most predominant (Ali et al., 1995; Prasanna et al., 2005). This fact is consistent with the number of unigenes for β-GAL obtained in this study.

Two β*-Glu* transcripts (comp43956, comp47526), which are involved in cellulose degradation, were up-regulated in ripe mango with 6 and 5-fold change, respectively. Expression analysis of β*-Glu* during ripening in fruits is very limited; most of the reports are about enzymatic analysis. In mango “Ataulfo,” the activity of this enzyme increased significantly during fruit ripening, which coincides with a dramatic decrease in firmness (Muy-Rangel et al., 2009). In sweet cherry (*Prunus avium* L.), the purification of one β-Glu has been reported and its activity increased during ripening (Gerardi et al., 2001).

In this work, an increase in the expression levels of five *EXP* transcripts (comp32472, comp51667, comp31446, comp32692, comp48981) was observed with 17, 5, 4, 2, and 2-fold changes, respectively. Only one mango *EXP* (comp42385) showed decreased expression (-2-fold change) in ripe fruit. In mango “Dashehari” and “Zill,” expansin genes had increased expression during fruit ripening (Sane et al., 2005; Zheng et al., 2012). *EXPA1* of mango “Dashehari” is 71% identical to *EXP* (comp51667) obtained in this study that also had increased expression levels in ripe fruit.

In mango, there is evidence that enzymatic hydrolysis of starch by α or β- amylases results in major loosening of the cell wall structure, which results in a decrease of the fruit firmness and increase of sweetness. Also, in mango “Kent” there is evidence that 90% of starch is catabolized in the early stages of fruit ripening, which is associated with fruit softening (Yashoda et al., 2006; Cárdenas-Coronel et al., 2012). In this work, four β-amylases encoding genes were found, two of them (comp44532, comp39992) were up-regulated (3, 2-fold changes), whereas the others did not changed in ripe mango. Meanwhile, three α-amylases were found (comp46096, comp40871, comp40434) highly expressed and did not change during ripening. In mango fruit, it has been shown that these genes have a very important role in fruit quality. Lima et al. (2001) reported that in fruit that showed lower activities of amylase, a spongy tissue was observed, which is a physiological disorder that affects the fruit quality and therefore, the potential for marketing.

#### Genes related to flavor quality

Fruit flavor is often dependent on aroma generated by volatile compounds and taste is associated with the sugar/acid ratio. Sucrose, glucose and fructose are the most abundant fruit sugars (Chaimanee and Suntornwat, 1994). In fruit ripening, accumulation of sucrose is due to the activity of the enzyme sucrose phosphate synthase (SPS) and sucrose phosphate phosphatase (SPP). In this study, six SPS genes were identified and they remained constantly expressed from mature-green to ripe mango. Two SPP genes were obtained and one of them (comp47111) was highly expressed in both stages. While seven sucrose synthase (SuSy) genes were identified, one of them (comp45134) was up-regulated and the rest did not changed in ripe mango. Recently, Islam et al. (2014) reported the presence of at least six SuSy genes in *Citrus*, four of them were expressed in fruit juice sacs and mature leaves and two expressed in young leaves. They demonstrated that the *Citrus* SuSy genes have different spatio-temporal expression patterns.

Mango fruit has more than 300 volatile compounds identified and terpene hydrocarbons (monoterpenes and sesquiterpenes) are the dominant volatile components (Pino et al., 2005). This transcriptome data set contained a total of 29 terpenoid backbone biosynthesis genes, and seven were DE (**Table 2**). Farnesyl pyrophosphate synthase (FPPS) catalyzes the synthesis of farnesyl pyrophosphate, a key intermediate in sterol and sesquiterpene biosynthesis. Recently, a *FPPS* gene was identified in mango “Alphonso” and it was expressed during fruit ripening (Kulkarni et al., 2013). An *FPPS* (comp48152) transcript was identified and showed a 2-fold increase in expression in ripe mango. Another important gene for aroma is the geranylgeranyl pyrophosphate synthase (GGPS), in this study two GGPS genes were identified, one of them (comp40639) was highly expressed in both stages and the other (comp48107) was DE (8-fold change) during ripening. In orange, mono and sesquiterpenes are the major components for flavor, while in climacteric varieties of melon, volatile esters are the predominant components together with sesquiterpenes, norisoprenoids, alcohols, and aldehydes (Sharon-Asa et al., 2003; El Hadi et al., 2013).

During ripening the membranes and the cell wall become permeable allowing the lipoxygenase (LOX) pathway to be active by sequential enzyme steps involving lipoxygenase and hydroperoxide lyase (HPL). Substrates for lipoxygenase, such as linoleic and linolenic acids, are common constituents of plant membranes (Riley et al., 1996). For mangos “Kensington Pride,” caprylic, capric, stearic, oleic, linoleic and linolenic acids were found in the pulp of ripe fruit (Lalel et al., 2003). Six LOX genes were found DE in this transcriptome in ripe mango; four genes (comp20603, comp47763, comp40188, comp44212) were up-regulated with 6, 5, 3, and 3-fold change, respectively. In tomato, at least five LOX genes have been reported, but there is only one (*TomloxC*) associated with volatile synthesis and it is expressed during tomato ripening (Heitz et al., 1997; Chen et al., 2004). This *TomloxC* is 47% identical with a LOX gene (comp44212) of mango.

#### Genes associated to color

Mesocarp in mango fruit changes from green to yellow-orange color due to the accumulation of carotenoids (Tanaka et al., 2008). It has been reported that all-*trans*-β-carotene, all-*trans*-violaxanthin and 9-*cis*-violaxanthin (as dibutyrates) are the principal “Ataulfo” mango carotenoids. During fruit ripening, carotenoids accumulate in an exponential manner, especially all-*trans*-β-carotene (Ornelas-Paz et al., 2008). In tomato, the genes encoding enzymes of the carotenoid pathway are regulated both transcriptionally (Klee and Giovannoni, 2011) and post-transcriptionally (Cazzonelli and Pogson, 2010). In this study, 10 genes that encode enzymes of carotenoid metabolism in mango mesocarp were DE; a PSY (comp42694) that carry out the first committed step, was highly expressed in mango and did not changed during ripening. While two identified phytoene desaturases (PDSs; comp46184 and comp44482) and one zeta-carotene desaturase (ZDS; comp39926) did not changed during ripening. However, a lycopene isomerase (*MiCRTISO*; comp42184) gene presented 4-fold change in ripe mango; similarly, a tomato *SlCRTISO* was also up-regulated at the red-ripe stage of fruit development (Smita et al., 2013).

In this study, a lycopene-beta-cyclase (*LCYB*; comp42574) transcript presented a -2 fold change in ripe mango. *LCYB* is 83% identical to persimmon fruit *DkLCYB* and relative expression levels of *DkLCYB* followed a similar expression pattern of mango *LCYB.* The levels of *DkLCYB* decreased during ripening and correlated with the increased contents of β-cryptoxanthin and β-carotene in this fruit (Zhao et al., 2011). Two carotenoid β-hydroxylase (CHYB1 and CHYB2) genes were identified in mango. *CHYB1* (comp45152) presented a 4-fold increase in ripe mango; while *CHYB2* did not changed during ripening. Zeaxanthin epoxidase (ZEP; comp41313) expression level was down-regulated in ripe mango with -2 fold change. Two additional genes from the isoprenoid pathway that may be associated with color development were identified in this transcriptome, unigene farnesyl diphosphate synthase (comp 48152) and GGPS (comp48107) were differentially expressed with 2 and 8-fold change, respectively.

### qRT-PCR VALIDATION

**FIGURE 4:**
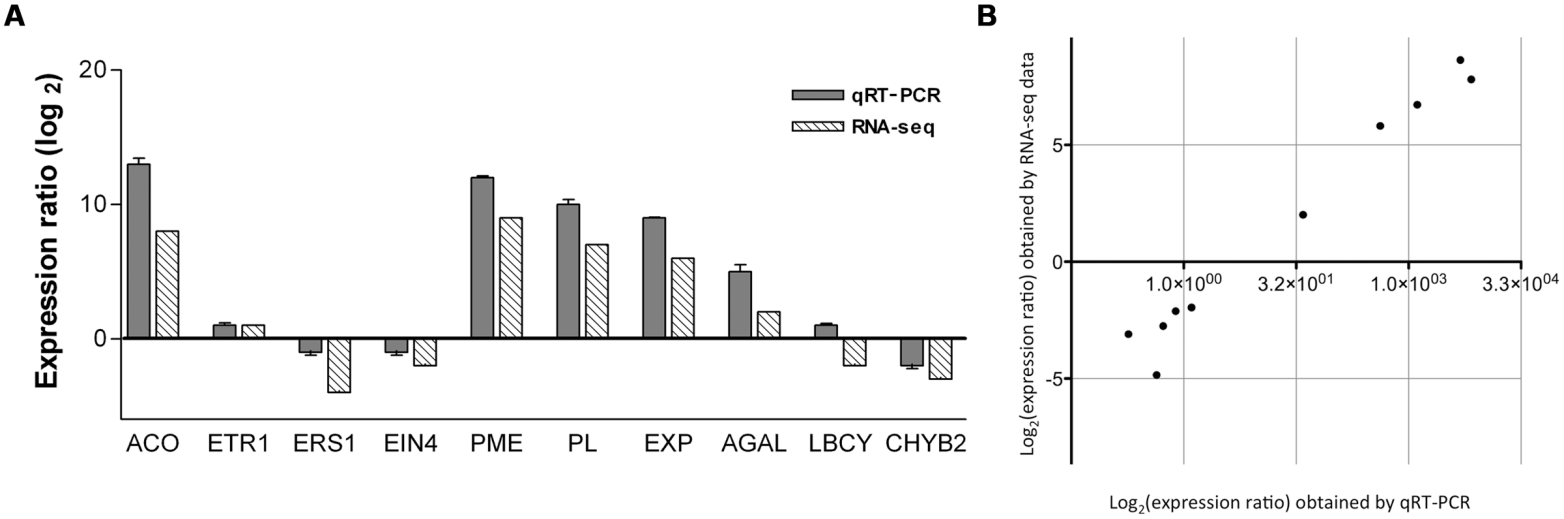
**(A)** Expression ratio (Log_2_) obtained by qRT-PCR and RNA-seq of 10 selected genes associated to fruit ripening. ACO, 1-aminocyclopropane-1-carboxylate oxidase (comp59876); ETR1, ethylene receptor (comp46286); ERS1, ethylene receptor sensor (comp32379); EIN4, ethylene insensitive 4 (comp34039); PME, pectin methyl esterase (comp45079); PL, pectate lyase (comp63384); EXP, expansin (comp51667); AGAL, alpha-galactosidase (comp46653); LCBY, lycopene beta-cyclase (comp42574); and CHYB2, carotenoid beta-hydroxylase 2 (comp39312). *GAPDH* was used as a reference gene for normalization of qRT-PCR data. Bars represent the error standard (*n* = 3). **(B)** Correlation between the gene expression ratios obtained from RNA-seq data and qRT-PCR. The RNA-seq Log_2_ value of the expression ratio is shown in the *y*-axis and the qRT-PCR Log_2_ value of the expression ratio in the *x-axis*.

In order to validate the DE data obtained by RNA-seq, the following single unigenes were selected to experimentally determine their expression levels by qRT-PCR: 1-aminocyclopropane-1-carboxylate oxidase (ACO) comp59876, ethylene receptor 1 (ETR1) comp46286, ethylene response sensor 1 (ERS1) comp32379, ethylene insensitive 4 (EIN4) comp34039, PME comp45079, PL comp63384, expansin (EXP) comp51667, alpha-galactosidase (AGAL) comp46653, lycopene beta-cyclase (LCYB) comp42574 and carotenoid beta-hydroxylase 2 (CHYB2) comp39312. The results for gene expression ratios between ripe and mature-green mango are shown in **Figure 4A**. A linear regression analysis showed an overall correlation coefficient of *R* = 0.97, which indicates a high correlation between transcript abundance assayed by qRT-PCR and the transcription profile revealed by RNA-seq data (**Figure 4B**). The results showed that although the exact fold changes of selected genes at the two data points (mature-green and ripe mango) varied between RNA-seq and qRT-PCR analyses, trends of gene expression by the two different approaches were consistent. The RNA-seq data reflects what is happening in the cell at the transcriptional level during mango ripening.

## CONCLUSION

The transcriptome of mango mesocarp captured most of the gene space in mango. We were able to identify many differentially expressed genes involved in ripening in mango. The expression data obtained by RNA-seq was favorably validated with the data obtained by qRT-PCR. Unigenes that code for gene products from pathways such as plant hormone signal transduction, starch and sucrose metabolism, galactose metabolism, terpenoid backbone biosynthesis and carotenoid biosynthesis, that allow characteristic changes during fruit ripening were identified in this transcriptome. Such genes can now be used to address particular questions in fruit ripening and allow expansion of the time course to earlier stages of fruit development.

## AUTHOR CONTRIBUTIONS

Conceived and designed the experiments: MAIO, SCF, MDC. Performed the experiments: MDC, ASF, CACV. Analyzed the data: MDC, ASF, AOL, CACV, MAPS, DNK. Contributed reagents/materials/analysis tools: MAIO, ASF, AOL, SCF. Wrote the paper: MAIO, MDC, MAPS, CACV, AOL, DNK.

## Conflict of Interest Statement

The authors declare that the research was conducted in the absence of any commercial or financial relationships that could be construed as a potential conflict of interest.
