# Supplementary material for: Mango (Mangifera indica L.) cv. Kent fruit mesocarp de novo transcriptome assembly identifies gene families important for ripening
Source: Front Plant Sci. 2015 Feb 18;6:62. doi: 10.3389/fpls.2015.00062 (PMC4332321; doi:10.3389/fpls.2015.00062)
Supplement: Supplementary file 1 [file Data_Sheet_1.PDF]

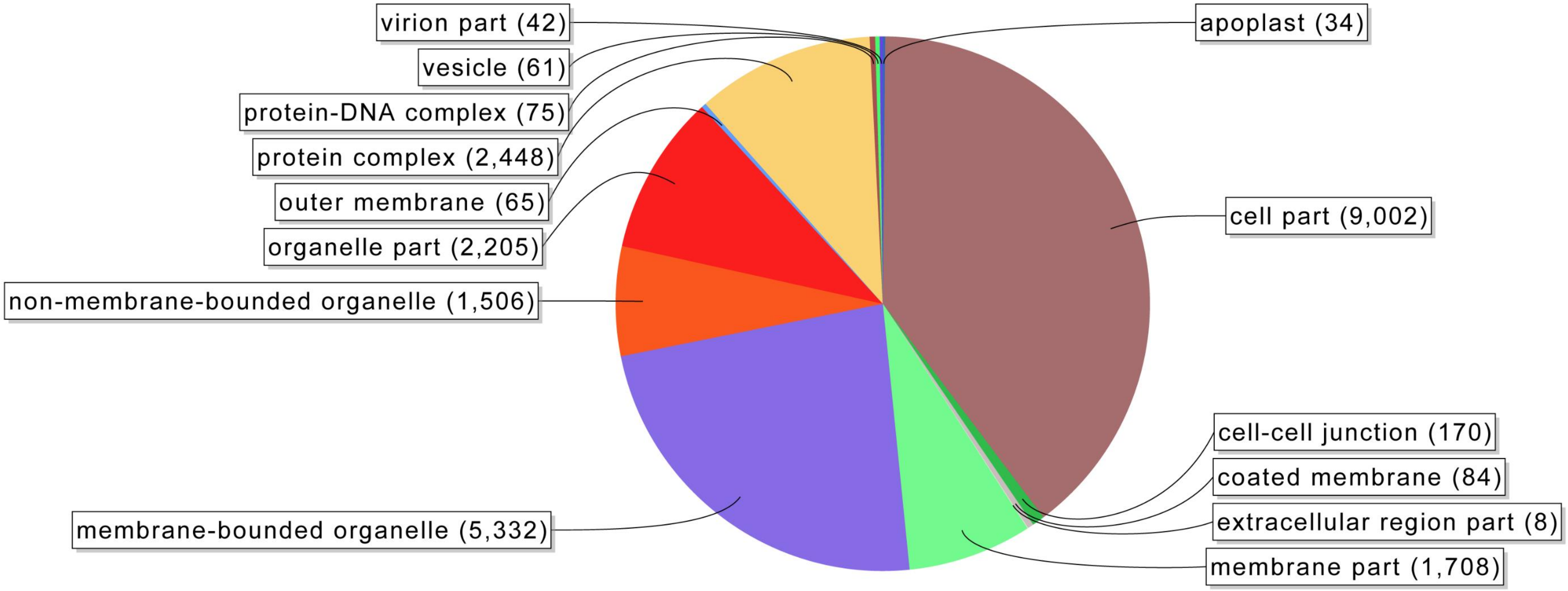

Supplementary Figure 1. Gene Ontology classification according to Cellular Component

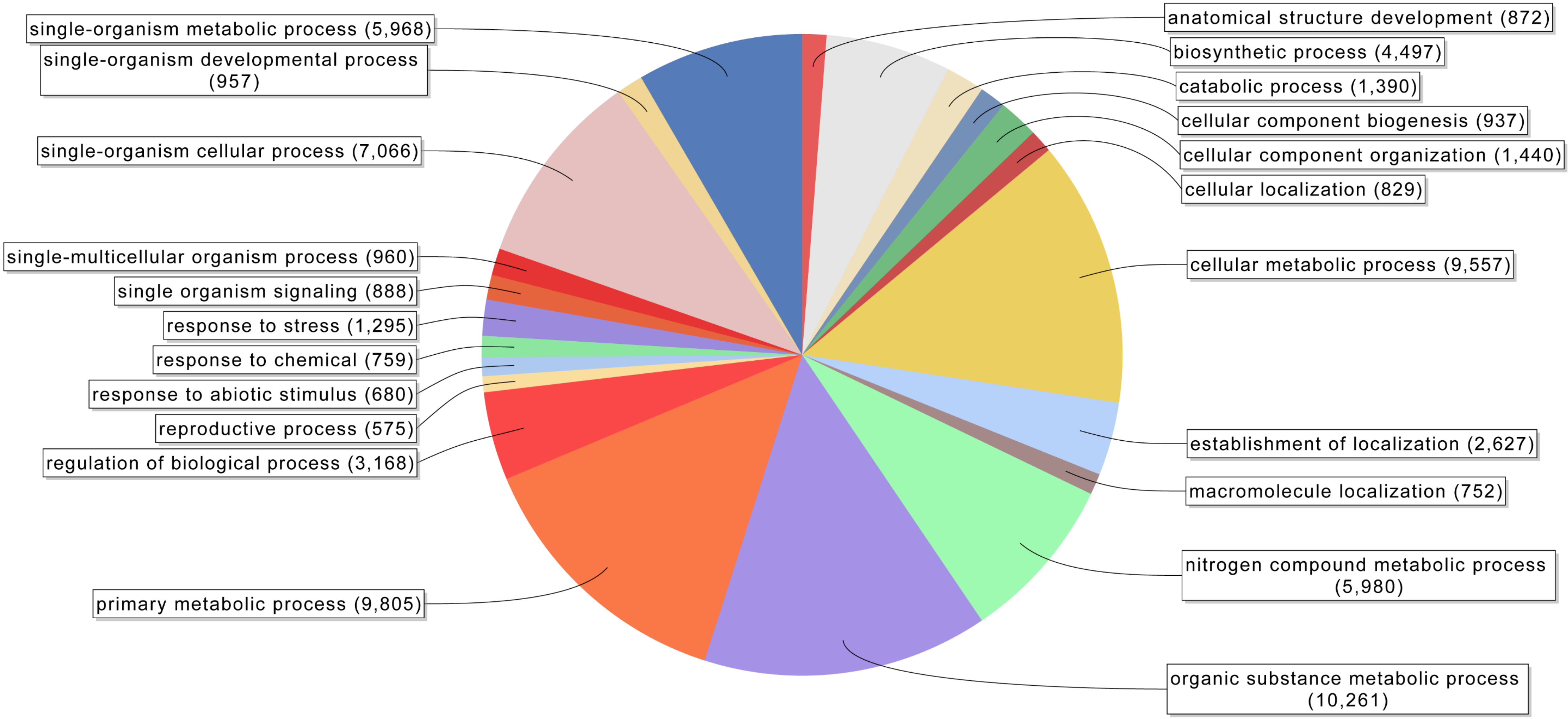

Supplementary Figure 2. Gene Ontology classification according to Biological Process

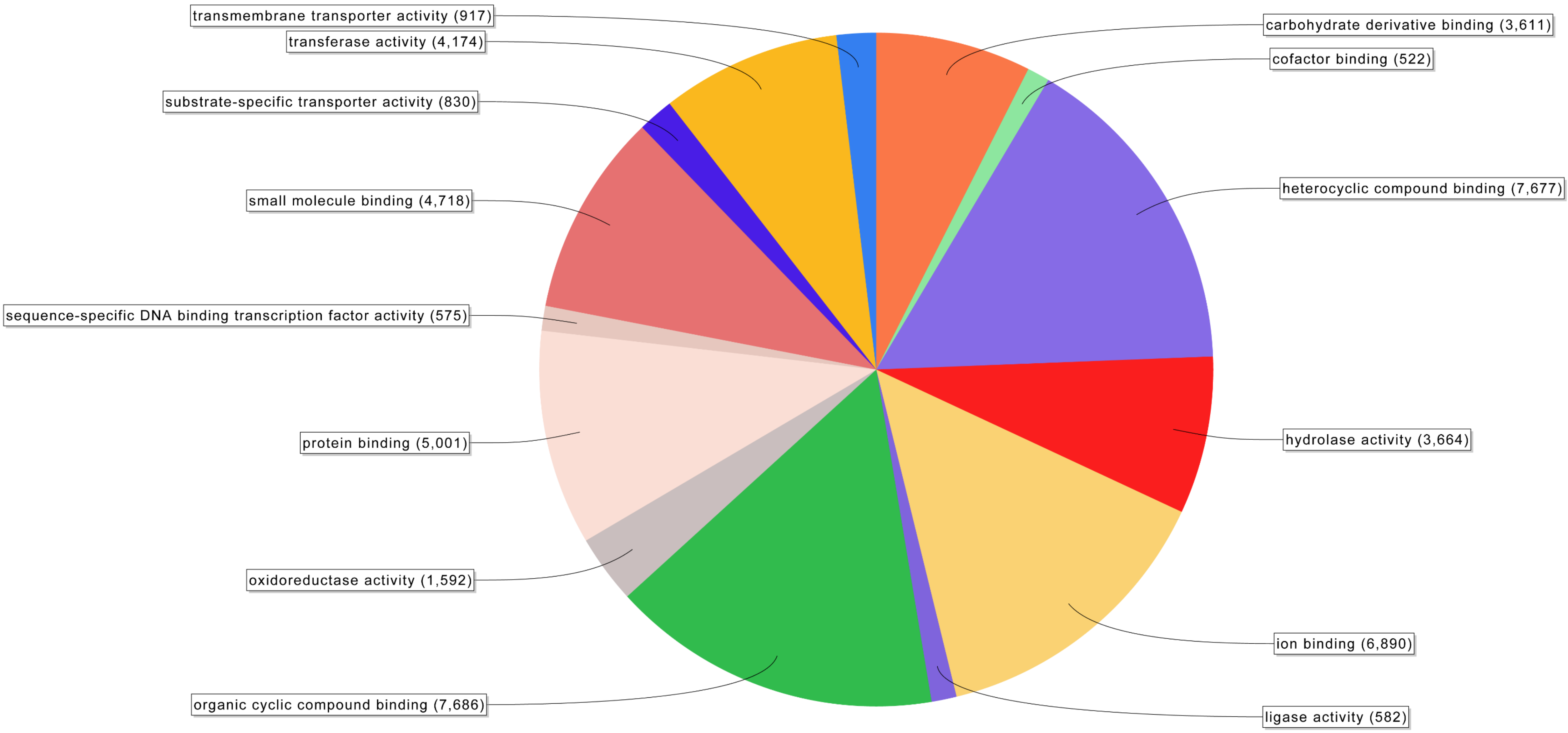

Supplementary Figure 3. Gene Ontology classification according to Molecular Function

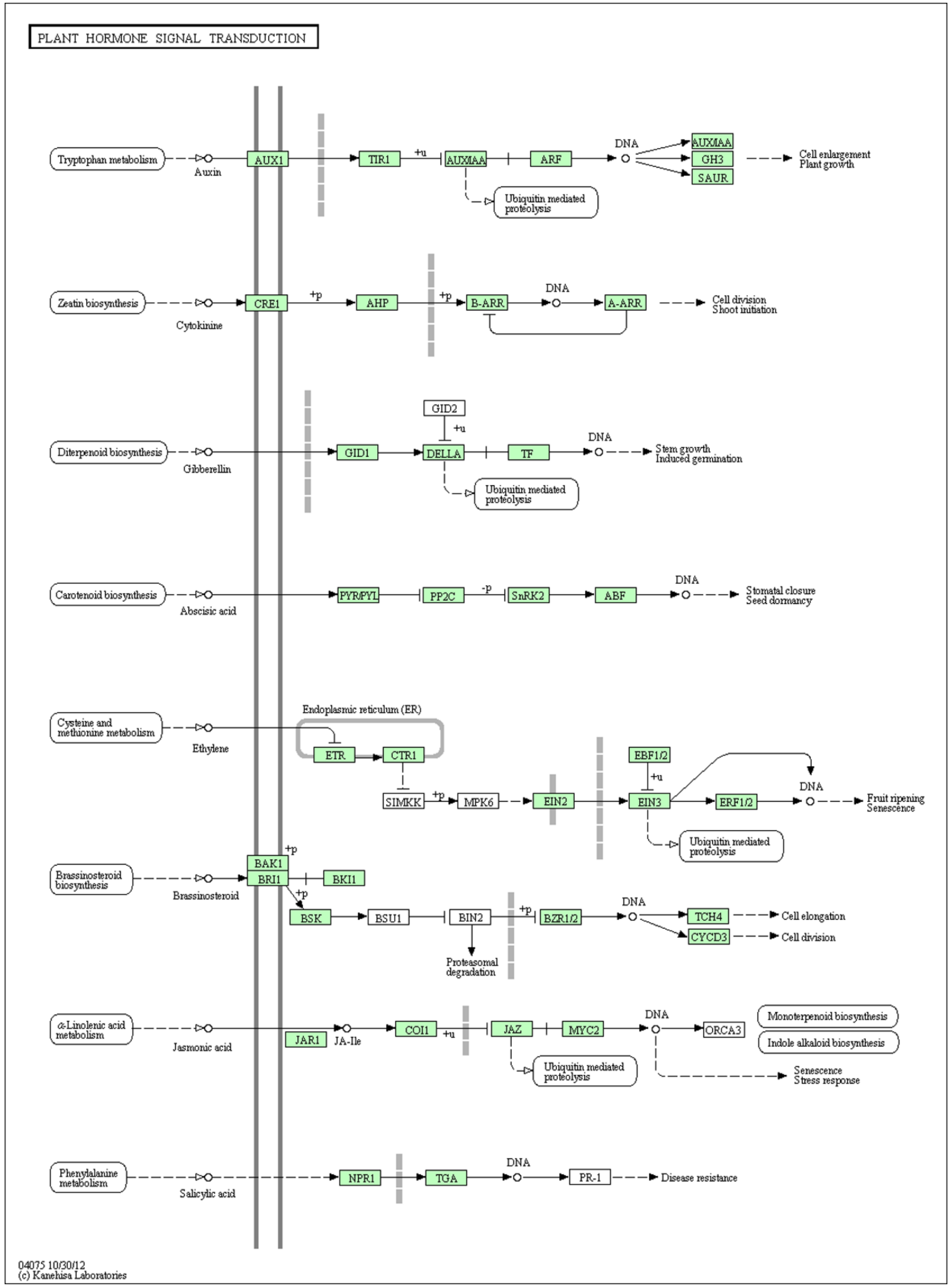

Supplementary Figure 4. KEGG metabolic pathway of plant hormone signal transduction obtained with mango Kent mesocarp transcriptome data.

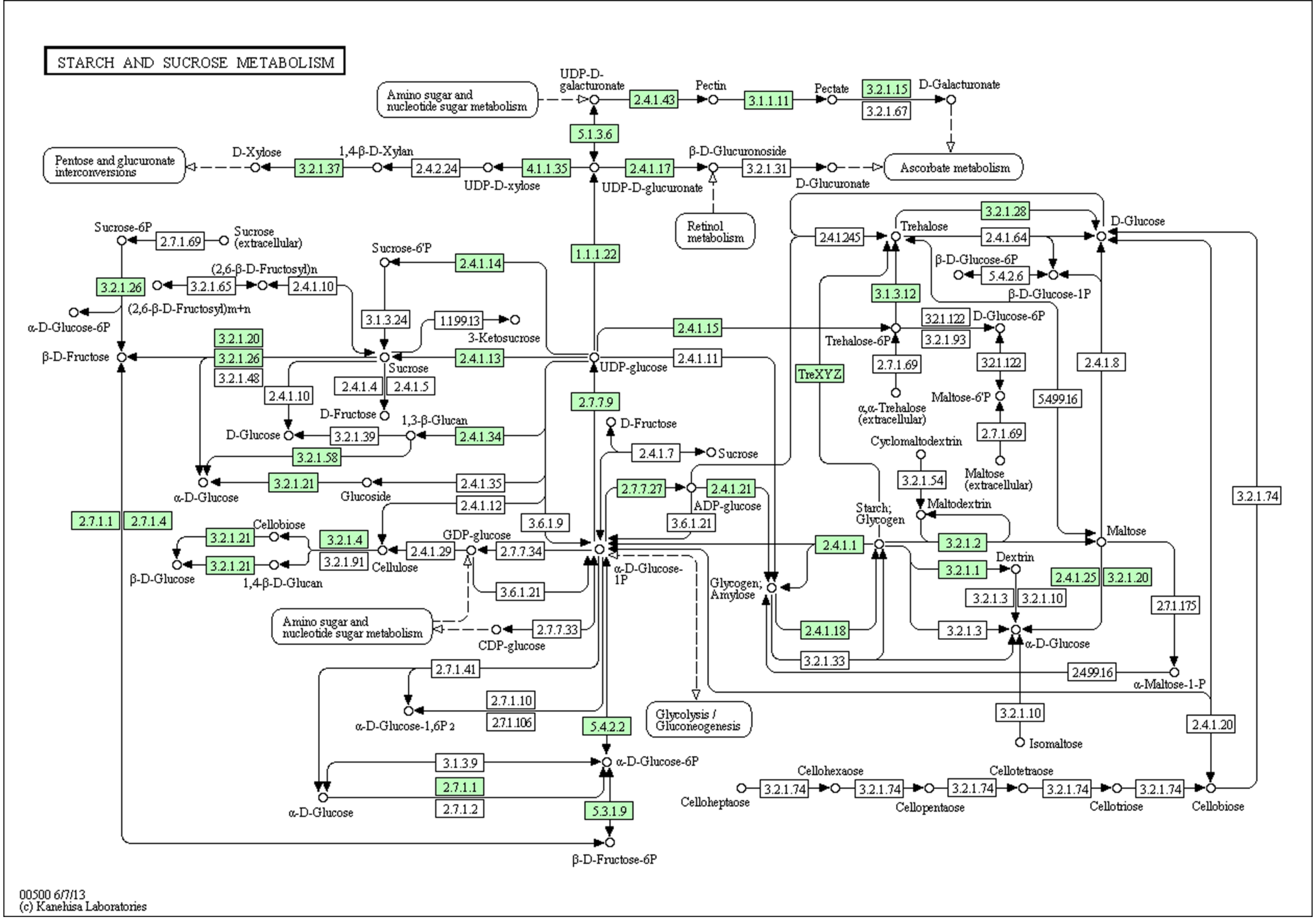

Supplementary Figure 5. KEGG metabolic pathway of terpenoid backbone biosynthesis obtained with mango Kent mesocarp transcriptome data.

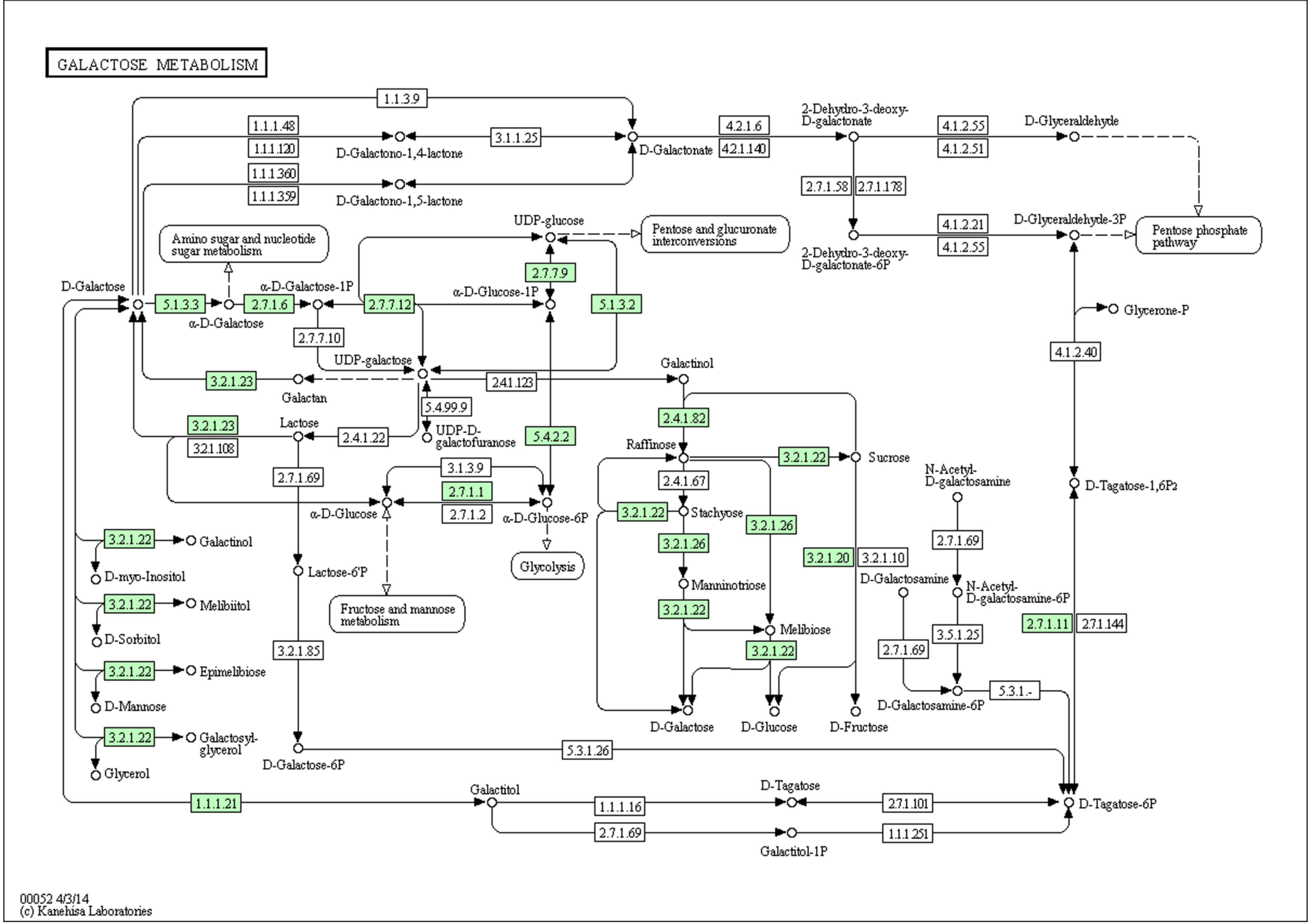

Supplementary Figure 6. KEGG metabolic pathway of galactose metabolism obtained with mango Kent mesocarp transcriptome data.

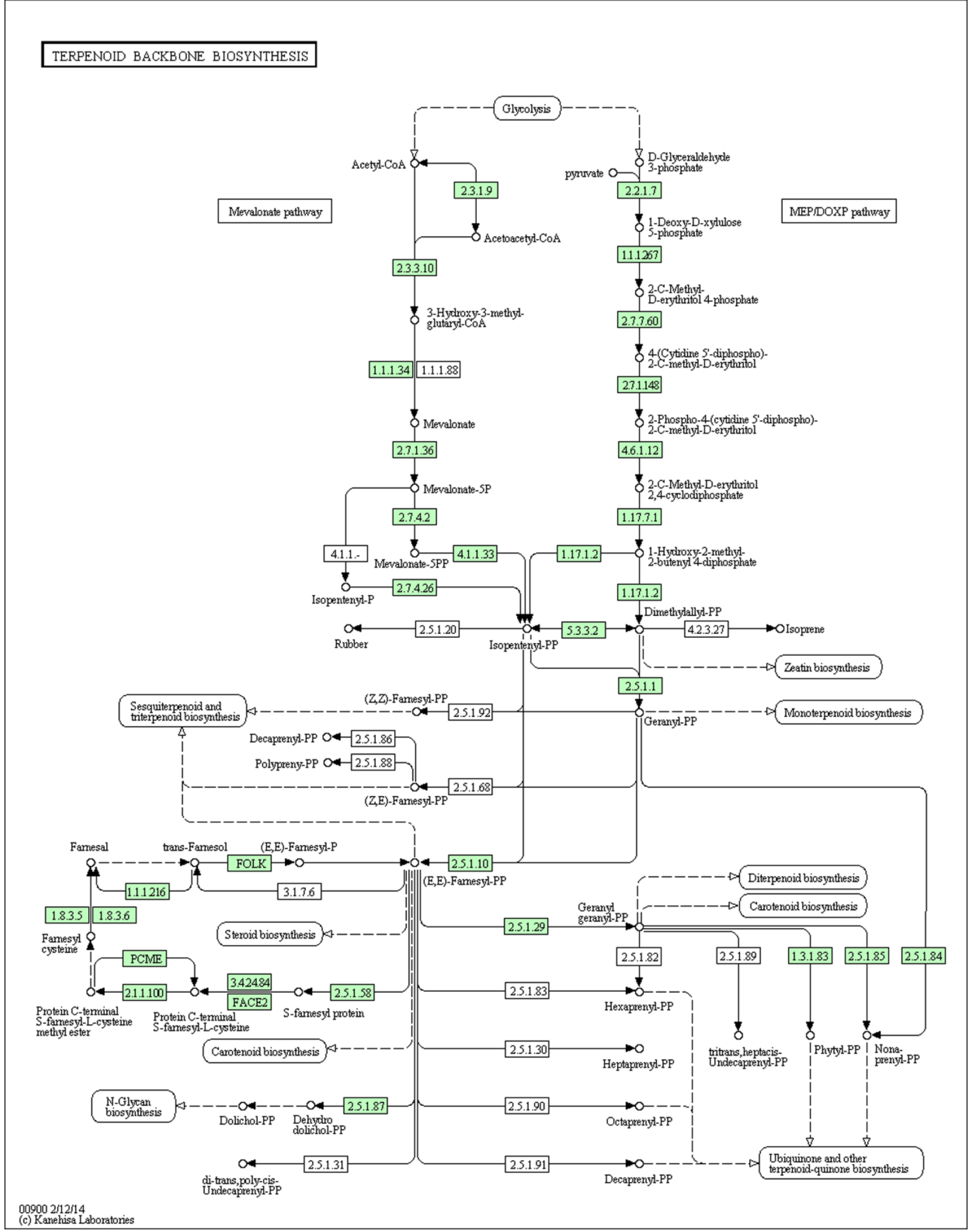

Supplementary Figure 7. KEGG metabolic pathway of terpenoid backbone biosynthesis obtained with mango Kent mesocarp transcriptome data.

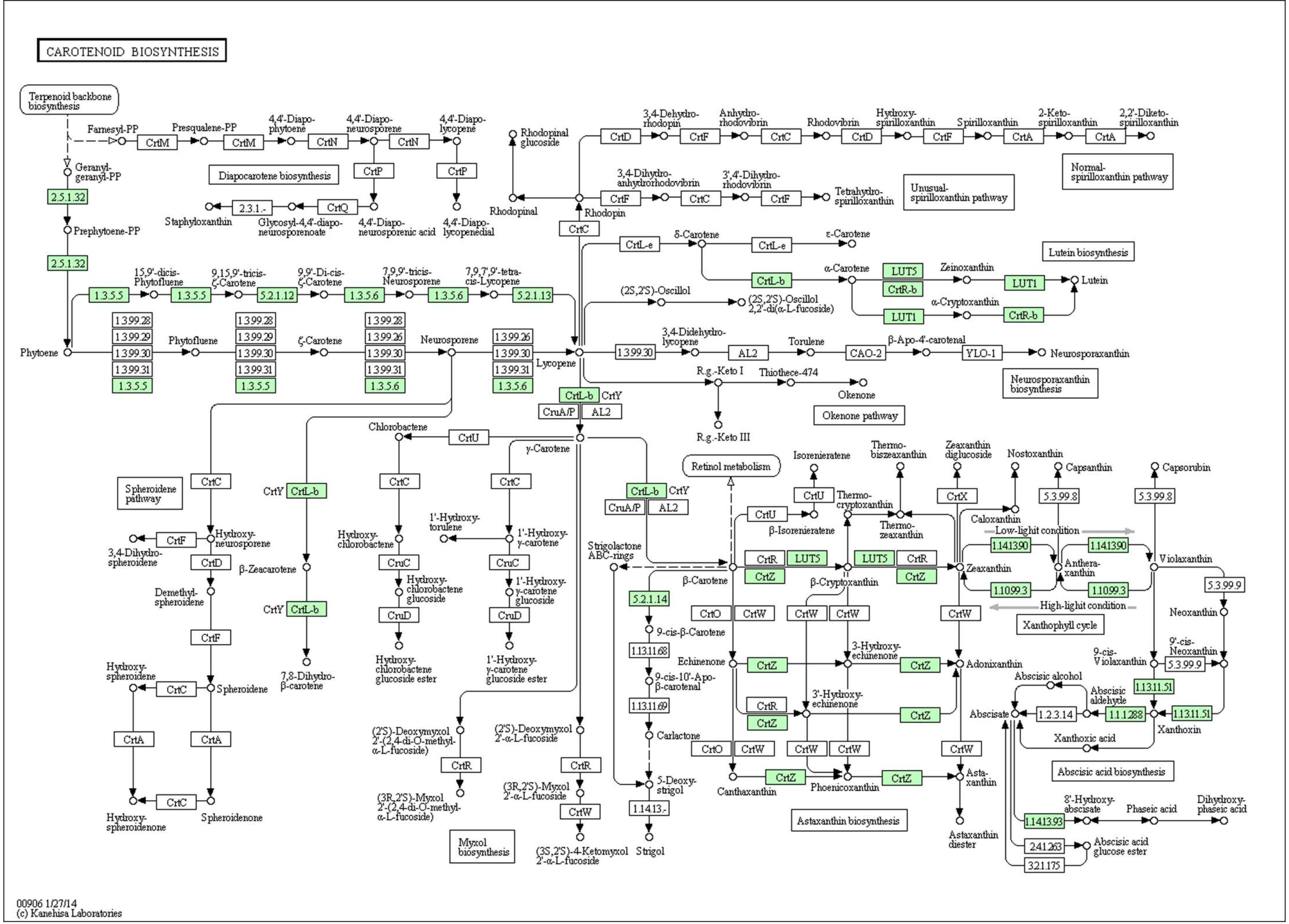

Supplementary Figure 8. KEGG metabolic pathway of carotenoid biosynthesis obtained with mango Kent mesocarp transcriptome data.

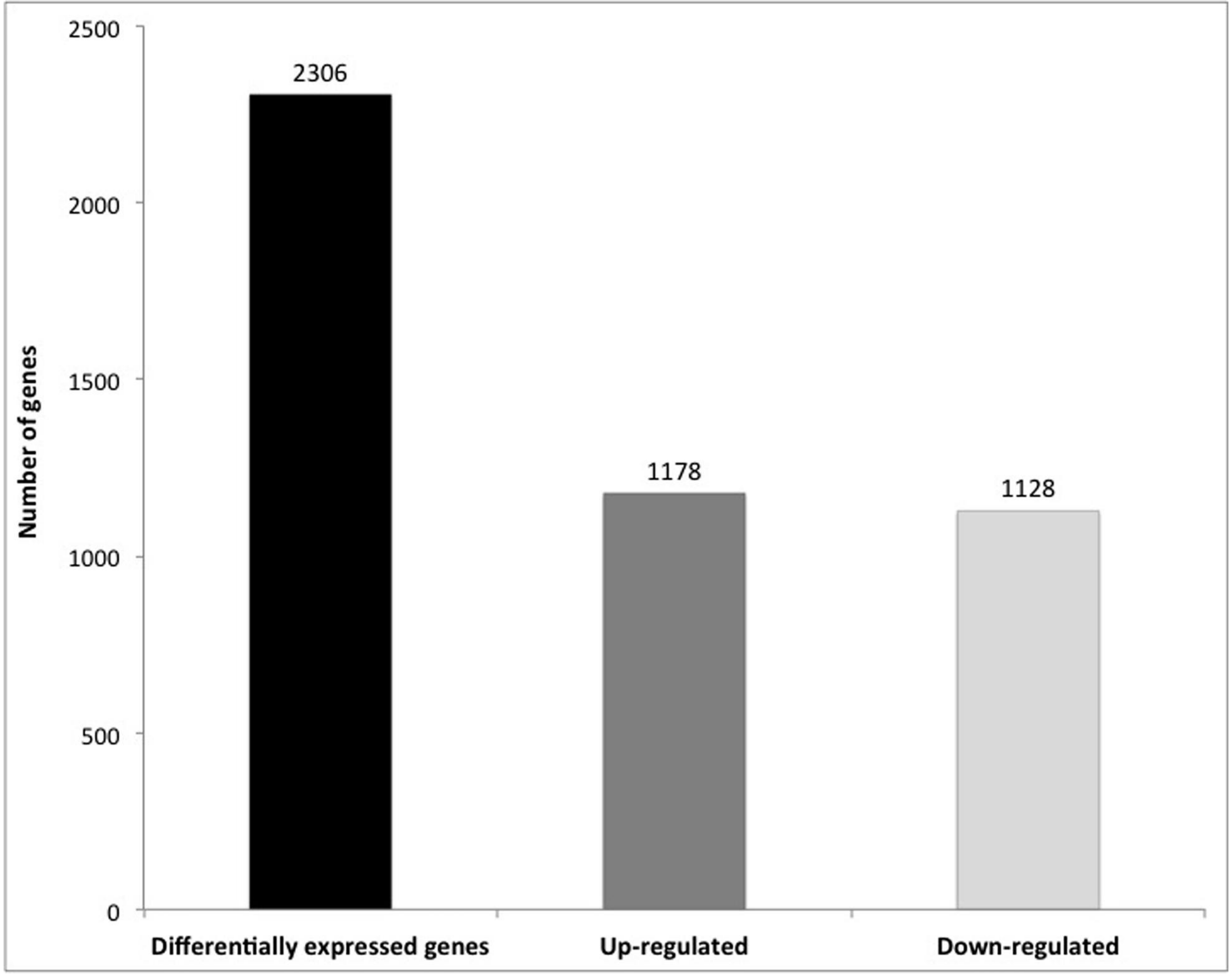

Supplementary Figure 9. Differential expressed genes in mango Kent mesocarp transcriptome
