# Supplementary material for: Mango (Mangifera indica L.) cv. Kent fruit mesocarp de novo transcriptome assembly identifies gene families important for ripening
Source: Front Plant Sci. 2015 Feb 18;6:62. doi: 10.3389/fpls.2015.00062 (PMC4332321; doi:10.3389/fpls.2015.00062)
Supplement: Supplementary file 2 [file Data_Sheet_2.PDF]

Supplementary Table 1. Primer sequences used for qRT-PCR validation of RNA-seq data

Gene	Forward primer (5' to 3')	Reverse primers (5' to 3')	Product size (bp)
EXP	GATCCAGGGAGACACCATTTAG	GCTTGTGGCTATGGAAACTTG	146
PME	GCAGCTTAGGAGGTGGAACAATC	TTAACTGGCCGAGCCAAATTCG	111
PL	TTGGGTTCACAGGGTCATAG	TTGCGCAATTGGGTTTGG	96
AGAL	CATGAGCATCCACACTAGCA	GGACCACTTGGACTTGAATCT	112
LBCY	GCTAGACTGAAGCACTTGGGC	CAGTACCGCCTATTCCAACTA	121
TPS	CAAGGAGGGACTTGACTATGT	CCTCTCTCCAGCTCATCCTTA	111
BCARHY	GGCACAGAAGAAGAACTTCTTT	CGAGTAGCTGGGACCTGAATA	133
EIN4	GGGTTTGAATGCCTTAGTGC	TTCCGGATTCTCATGGCTAC	113
ERS1	AATGGCAAGTCCAAGTCCTG	ATGCAATGGCCACTTCTACC	151
ETR1	TTGCAGTTCGTGTTCCACTC	TCAGCAACCACTTCAACGAG	160
ACCO	GTTGGTGACTTGGACTGG	GCCCTAGATTCTCACAGAGC	156

Supplementary Table 2. Top 30 of metabolic pathways of genes in total mango transcriptome based on Kyoto Encyclopedia of Genes and Genomes (KEGG)

Top	KEGG Pathway	Number of genes
1	Biosynthesis of amino acids	96
2	Ribosome	95
3	RNA transport	93
4	Spliceosome	90
5	Purine metabolism	82
6	Carbon metabolism	77
7	Protein processing in endoplasmic reticulum	75
8	Pyrimidine metabolism	68
9	Oxidative phosphorylation	59
10	Ubiquitin mediated proteolysis	55
11	Ribosome biogenesis in eucaryotes	54
12	Cell cycle	49
13	mRNA surveillance pathway	48
14	Cell cycle - yeast	45
15	RNA degradation	44
16	Arginine and proline metabolism	38
17	Plant hormone signal transduction	37
18	Endocytosis	37
19	Amino sugar and nucleotide sugar metabolism	36
20	Nucleotide excision repair	35
21	Peroxisome	34
22	Glycerophospholipid metabolism	34
23	Meiosis - yeast	33
24	Proteasome	33
25	Starch and sucrose metabolism	32
26	Glycine, serine and threonine metabolism	32
27	Cysteine and methionine metabolism	32
28	Photosynthesis	31
29	Glycolysis / Gluconeogenesis	30
30	Basal transcription factors	30

Supplementary Table 3. Top 30 of metabolic pathways of genes differentially expressed (FDR<0.05) based on Kyoto Encyclopedia of Genes and Genomes (KEGG)

Top	KEGG Pathway	Number of genes
1	Carbon metabolism	20
2	Plant hormone signal transduction	20
3	Biosynthesis of amino acids	18
4	Starch and sucrose metabolism	12
5	Fatty acid metabolism	12
6	Glycolysis / Gluconeogenesis	12
7	Arginine and proline metabolism	11
8	Protein processing in endoplasmic reticulum	11
9	Plant-pathogen interaction	11
10	Pyruvate metabolism	10
11	2-Oxocarboxylic acid metabolism	9
12	Photosynthesis	9
13	Amino sugar and nucleotide sugar metabolism	7
14	Oxidative phosphorylation	7
15	Photosynthesis - antenna proteins	7
16	Carbon fixation in photosynthetic organisms	7
17	Fatty acid biosynthesis	7
18	Fatty acid degradation	7
19	Cysteine and methionine metabolism	
20	Valine, leucine and isoleucine degradation	7
21	Terpenoid backbone biosynthesis	7
22	Flavonoid biosynthesis	7
23	alpha-Linolenic acid metabolism	6
24	Biosynthesis of unsaturated fatty acids	6
25	Phenylalanine metabolism	6
26	Phenylalanine, tyrosine and tryptophan biosynthesis	6
27	beta-Alanine metabolism	6
28	Carotenoid biosynthesis	6
29	Phenylpropanoid biosynthesis	6
30	Ubiquitin mediated proteolysis	6
